# Pressure-induced iso-structural phase transition and metallization in WSe_2_

**DOI:** 10.1038/srep46694

**Published:** 2017-05-04

**Authors:** Xuefei Wang, Xuliang Chen, Yonghui Zhou, Changyong Park, Chao An, Ying Zhou, Ranran Zhang, Chuanchuan Gu, Wenge Yang, Zhaorong Yang

**Affiliations:** 1 Anhui Key Laboratory of Condensed Matter Physics at Extreme Conditions, High Magnetic Field Laboratory, CAS and University of Science and Technology of China, Hefei 230031, China; 2 High Pressure Collaborative Access Team, Geophysical Laboratory, Carnegie Institution of Washington, Argonne, Illinois 60439, USA; 3 Center for High Pressure Science and Technology Advanced Research (HPSTAR), Shanghai 201203, China; 4 High Pressure Synergetic Consortium (HPSynC), Geophysical Laboratory, Carnegie Institution of Washington, Argonne, Illinois 60439, USA; 5 Key Laboratory of Materials Physics, Institute of Solid State Physics, Chinese Academy of Sciences, Hefei 230031, China; 6 Collaborative Innovation Center of Advanced Microstructures, Nanjing University, Nanjing 210093, China

## Abstract

We present *in situ* high-pressure synchrotron X-ray diffraction (XRD) and Raman spectroscopy study, and electrical transport measurement of single crystal WSe_2_ in diamond anvil cells with pressures up to 54.0–62.8 GPa. The XRD and Raman results show that the phase undergoes a pressure-induced iso-structural transition via layer sliding, beginning at 28.5 GPa and not being completed up to around 60 GPa. The Raman data also reveals a dominant role of the in-plane strain over the out-of plane compression in helping achieve the transition. Consistently, the electrical transport experiments down to 1.8 K reveals a pressure-induced metallization for WSe_2_ through a broad pressure range of 28.2–61.7 GPa, where a mixed semiconducting and metallic feature is observed due to the coexisting low- and high-pressure structures.

Band structure engineering in transition metal dichalcogenides MX_2_ (M = Mo, W; X = S, Se) has attracted considerable interest due to not only the resulted exotic physics but also their potential technological relevances^1^,^2^,^3^,^4^,^5^,^6^,^7^,^8^,^9^,^10^,^11^,^12^,^13^,^14^. The MX_2_ structure consists of X-M-X sandwiched monolayers linked by weak van der Waals (vdW) interlayer forces, as shown in Fig. 1(a). When mechanically exfoliated, the indirect gap of bulk MX_2_ increases and transforms to a direct gap in the monolayer limit^1^, making them appealing for applications in electronics, optoelectronics as well as valleytronics^2^,^3^,^4^,^5^,^6^. In addition, doping^7^, temperature^8^ and strain^1^,^9^,^10^ can also be used to manipulate the size and nature of the gap and to drive a semiconducting to metal transition. Moreover, by intercalation or an electrostatic carrier injection using a field effect transistor, the carrier mobility can be modulated and even superconductivity can be induced^11^,^12^,^13^,^14^.

Pressure is known as a powerful tool to tune lattice and the band gap, and its related studies on semiconducting MX_2_, indeed, have given rise to many novel physical phenomena. For example, MoS_2_ was reported to undergo a pressure-induced semiconductor to metal transition at high pressure^15^,^16^. Under further compression up to megabar, even a superconductivity is observed^17^. The pressure-induced metallization in MoS_2_ was related to a 2*H*_*c*_ to 2*H*_*a*_ iso-structural transition via layer sliding^15^,^16^,^18^. Very recently, the tunable electronic band gap of MoS_2_ was found to correlate intrinsically to a common structural characteristic, which was proposed to be readily extendible to other semiconducting MX_2_^19^.

The pressure-induced metallization may not be always accompanied by a structural transition. In MoSe_2_, a pressure-induced metallization is observed, without any sign of structural change. The absence of structural transition in MoSe_2_ is empirically related to the relatively delocalized 4*p* orbitals of Se^2−^ than the 3*p* orbitals of S^2−^, which results in stronger interlayer interactions to prevent the sliding process and thus the structural transition^20^.

To date, the high-pressure properties of WSe_2_ have not been well understood due to the lack of sufficient experimental data. Liu *et al*. found a slope change of electrical resistivity at ~38.1 GPa for WSe_2_, but the data was taken only in a temperature range of 290–420 K^21^. Based on theoretical calculations, this resistivity anomaly is related to a semiconductor to semimetal transition^21^. An early XRD study showed that the pristine 2*H*_*c*_ structure of WSe_2_ was stable up to 35.8 GPa^22^. Later, a Raman investigation confirmed no structural anomaly up to 20 GPa^23^. Recently, a first-principles study revealed an anomaly at 40 GPa consistently in the bulk modulus, Young’s modulus and Poisson’s ratio of WSe_2_, which may imply a possible structural transition^21^,^24^. More experimental efforts are therefore necessary to gain comprehensive knowledge of the high-pressure behavior of WSe_2_.

In the present study, we employ combined *in situ* synchrotron XRD and Raman spectroscopy, and electrical transport measurements to study the structural and electronic properties of single crystal WSe_2_. Both the XRD and the Raman data document an iso-structural transition. The resistivity measurements down to 1.8 K indicate that a pressure-induced metallization occurs in a broad pressure range, due to the coexistence of the low-pressure and high-pressure structural phases.

## Results and Discussion

**Figure 1:**
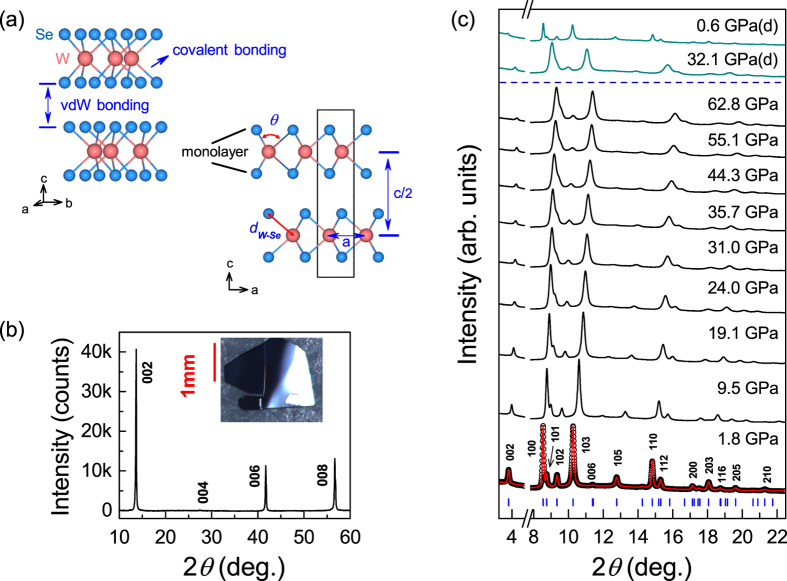
(**a**) The atomic arrangement of WSe_2_ in perspective and side views. W is represented by pink balls and Se by blue balls. *a* and *c* are cell parameters. *θ* is Se-W-Se bond angle and *d*_*W*_ _−_ _*Se*_ is W-Se bond length. (**b**) The XRD pattern of WSe_2_ at ambient conditions. Inset: a piece of WSe_2_ single crystal. (**c**) Representative synchrotron X-ray diffraction patterns of WSe_2_ during the compression and decompression (by d) runs (*λ* = 0.4246 Å). The observed and Rietveld refined profiles at 1.8 GPa is shown. The open circles and red lines are the experimental and calculated data. The positions of the Bragg reflections are marked by vertical sticks. The refined value is *R*_*P*_ = 1.05% with weighted profile *R*_*WP*_ = 1.40%.

**Figure 2:**
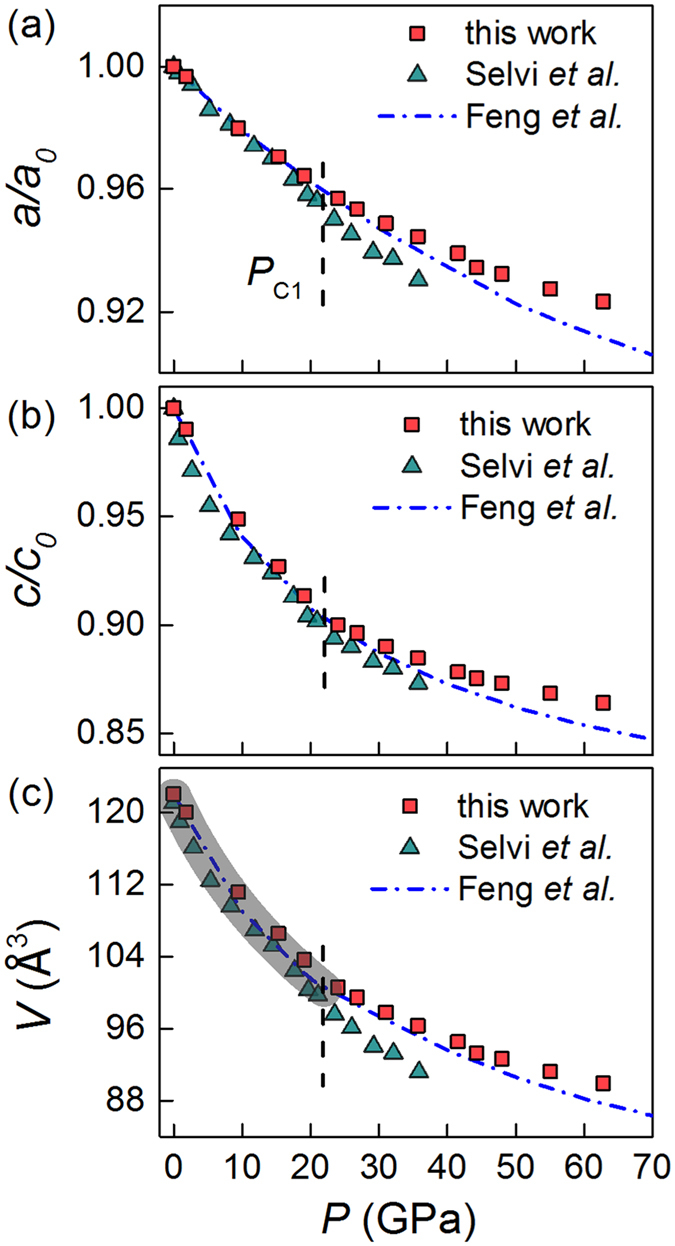
The normalized cell parameters *a/a*_0_ (**a**), *c/c*_0_ (**b**), and the volume *V* (**c**) as a function of pressure. The experimental data points from Selvi *et al*.^22^ and the theoretical results of Feng *et al*.^24^ are plotted for comparison. The gray zone represents the fitted data with the third-order Birch-Murnaghan equation of states.

High-quality plate-like single crystals (inset of Fig. 1(b)) with typical dimensions of 2 mm × 1 mm × 0.2 mm were grown by chemical vapor transport method^25^,^26^. The crystal structure was characterized by single crystalline XRD (Fig. 1(b)), which shows a (00*l*) preferable orientation (defined as c axis). At ambient conditions, WSe_2_ crystallizes in a hexagonal lattice with space group of *P*6_3_/*mmc*^27^, referred to as 2*H*_*c*_-WSe_2_. To enrich the knowledge of the high-pressure structural symmetry than a previous study to 35.8 GPa^22^, we carried out *in situ* synchrotron XRD measurements by extending the pressure up to 62.8 GPa at room temperature. Selected experimental patterns are displayed in Fig. 1(c). At 1.8 GPa, the XRD profile is well reproduced by the 2*H*_*c*_ structure (see the bottom of Fig. 1(c)). Upon compression, there seems no evident anomaly throughout the whole pressure range studied except for a consistent broadening of all peaks above 19.1 GPa, which is in good agreement with a previous report to 35.8 GPa^22^. Assuming that the pristine 2*H*_*c*_ structure of WSe_2_ exists up to 62.8 GPa, we calculated the normalized cell parameters *a/a*_0_ and *c/c*_0_ as well as the volume *V* as a function of pressure. The results are displayed in Fig. 2. For comparison, previous theoretical and experimental structural data^22^,^24^ are also included. It is observed that, below 20 GPa (marked as *P*_*C*1_), all data agrees nicely with each other. Above *P*_*C*1_, however, a deviation starts to appear. The deviation above *P*_*C*1_ between the present and previous data may be related to a structural transition since all of these XRD data was fitted only based on a 2*H*_*c*_ structural model, without considering the possibility of a structural transition induced by pressure. In addition, here the broadening of all XRD peaks above 19.1 GPa may be a manifestation of this pressure-induced structural anomaly. We fitted the pressure-volume data below *P*_*C*1_ using the third-order Birch-Murnaghan equation of state^28^ (as shown by a gray zone in Fig. 2(c)). The bulk modulus is estimated to be B_0_ = 72(5) GPa and its first pressure derivative 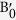 = 4.6(5).

**Figure 3:**
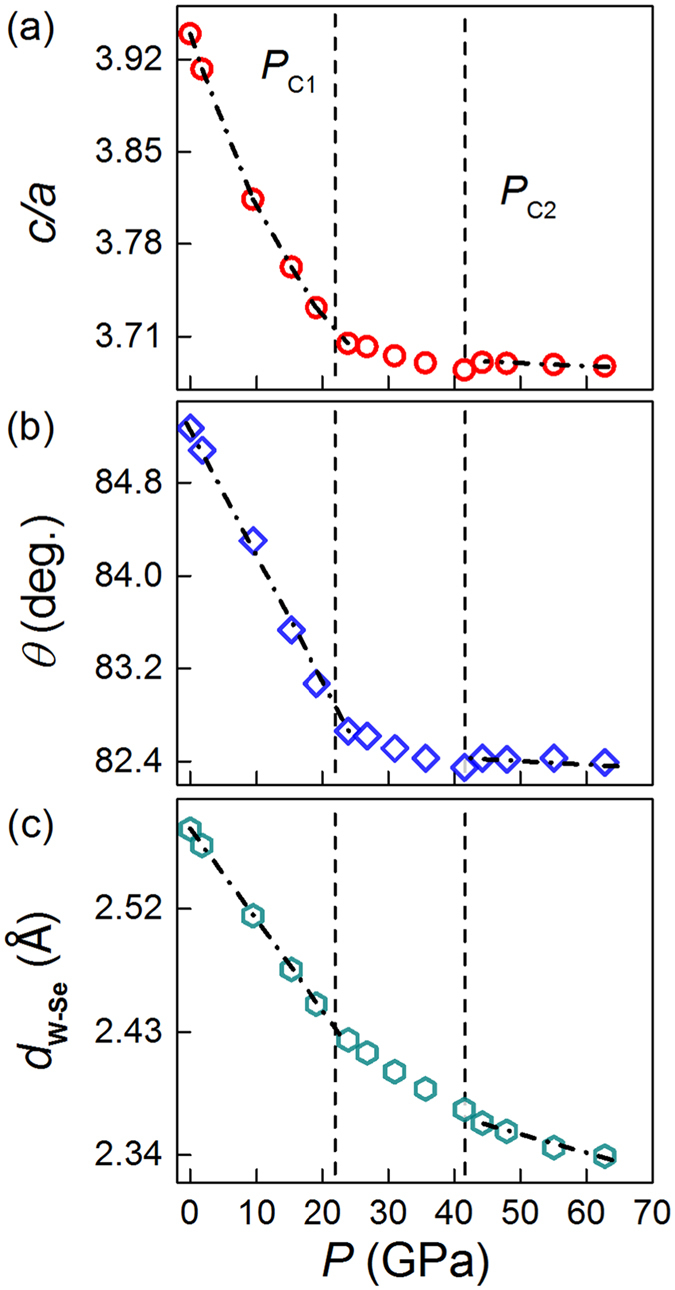
Pressure dependence of (**a**) axial ratio *c/a*, (**b**) *θ*, and (**c**) *d*_*W*_ _−_ _*Se*_ of WSe_2_. The error bars, given by GSAS, are smaller than the size of the markers. The lines are for eye guide.

To further check whether there is a possible structural transition, we plotted the axis ratio (*c/a*), the Se-W-Se bond angle (*θ*) and the W-Se bond length (*d*_*W*_ _−_ _*Se*_) as a function of pressure (as illustrated in Fig. 1(a)). From Fig. 3(a), *c/a* shows a nonlinear decrease during the initial compression, indicating that the cell parameter *c* is much more compressible than *a*. This can be related to the weaker vdW interlayer forces relative to the stronger intralayer covalent chemical bonding^29^. Above *P*_*C*1_, the change rate of *c/a* varies drastically, following a roughly linear decrease with pressure, which suggests that the cell parameters *a* and *c* have nearly-isotropic contractions. Further compression to about 40 GPa (marked as *P*_*C*2_), the change rate of *c/a* shows a small uprise and then decreases almost linearly up to the highest pressure. At 62.8 GPa, *a* and *c* are reduced by 7.5% (Fig. 2(a)) and 13.4% (Fig. 2(b)), respectively, which clearly evidences an anisotropic compression. Meanwhile, both the *θ* and *d*_*W*_ _−_ _*Se*_ also display two anomalies with increasing pressure, namely, changes of slope at *P*_*C*1_ and *P*_*C*2_ (Fig. 3(b) and (c)). In addition to the above mentioned broadening of peaks (Fig. 1(c)) and deviation behavior (Fig. 2(c)), the consistent discontinuities in *c/a, θ* and *d*_*W*_ _−_ _*Se*_ at *P*_*C*1_ and *P*_*C*2_ should imply structural changes. It is noted that similar discontinuities in *c/a* or volume were also observed in high pressure MoS_2_, which was related to an iso-structural transition from 2*H*_*c*_ to 2*H*_*a*_^15^. The iso-structural transition involves only a lateral sliding of adjacent layers and the change is subtle, probably making it not easy to decipher the structural changes directly from the raw XRD data.

**Figure 4:**
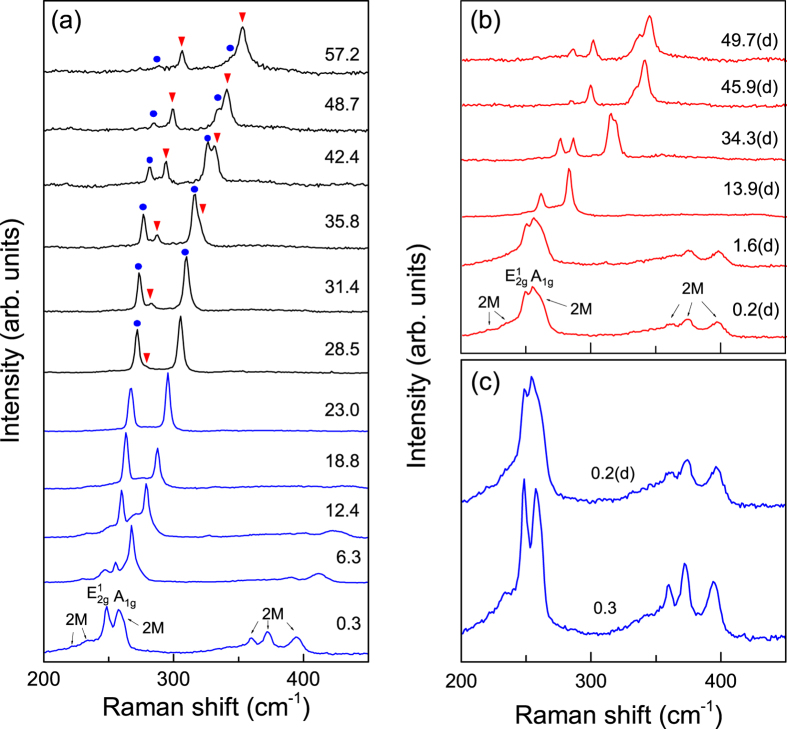
Room temperature Raman spectra of WSe_2_ at selected pressures up to 57.2 GPa in the compression (**a**) and decompression (denoted by d) (**b**) runs. The numbers represent pressures in unit of GPa. The blue solid dots and red triangles indicate the appearance of splitting of the 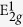 and A_1*g*_ modes owing to pressure-induced phase transitions. (**c**) Comparison of Raman spectra at 0.3 and 0.2 GPa during compression and decompression, respectively.

Raman spectroscopy is a sensitive and effective technique in detecting small changes in the structural transition. We carried out a detailed Raman study with pressures up to 57.2 GPa. Selected Raman spectra during compression and decompression are displayed in Fig. 4. Under ambient conditions, two main and several small Raman peaks are observed in the frequency range of 200–450 cm^−1^. The peaks at 248.4 and 257.3 cm^−1^ can be assigned to the 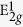 and A_1*g*_ vibrational modes respectively, and other small peaks (2 *M* modes) are related to the second order and combinational Raman modes, consistent with previous literature results^23^,^30^,^31^. As the pressure is increasing, while the peak intensity of A_1*g*_ shows only a small variation, the peak intensity of the 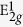 mode increases significantly. Going up from 28.5 GPa, the peak intensity of 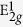 reduces rapidly and the 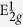 mode begins to split into two peaks. Subsequently, a splitting of A_1*g*_ mode starts to appear at 35.8 GPa. The splitting of the 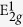 and A_1*g*_ Raman modes for WSe_2_ under pressure is also reproduced in another run (see supplementary materials for details). Both the change of peak intensity and splitting of peaks give strong evidences of a pressure-induced iso-structural transition like in MoS_2_^16^. Further increasing the pressure up to the highest pressure of 57.2 GPa, the splittings of 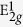 and A_1*g*_ can still be clearly observed, indicating an incompleteness of the structural phase transition and coexistence of the low- and high-pressure structural phases up to this pressure. In addition, we noted that the intensity of both Raman peaks from the high-pressure phase (red triangles, Fig. 4(a)) becomes dominant over those from the low-pressure phase (blue solid dots, Fig. 4(a)) above around 40 GPa, in excellent agreement with the critical pressure *P*_*C*2_ observed in our above XRD results (Fig. 3). When the pressure is released, the Raman spectra first show a splitting in the intermediate pressure (Fig. 4(b)) and then came back (0.2 GPa) to the starting positions at 0.3 GPa (Fig. 4(c)), manifesting the reversible structural transition.

**Figure 5:**
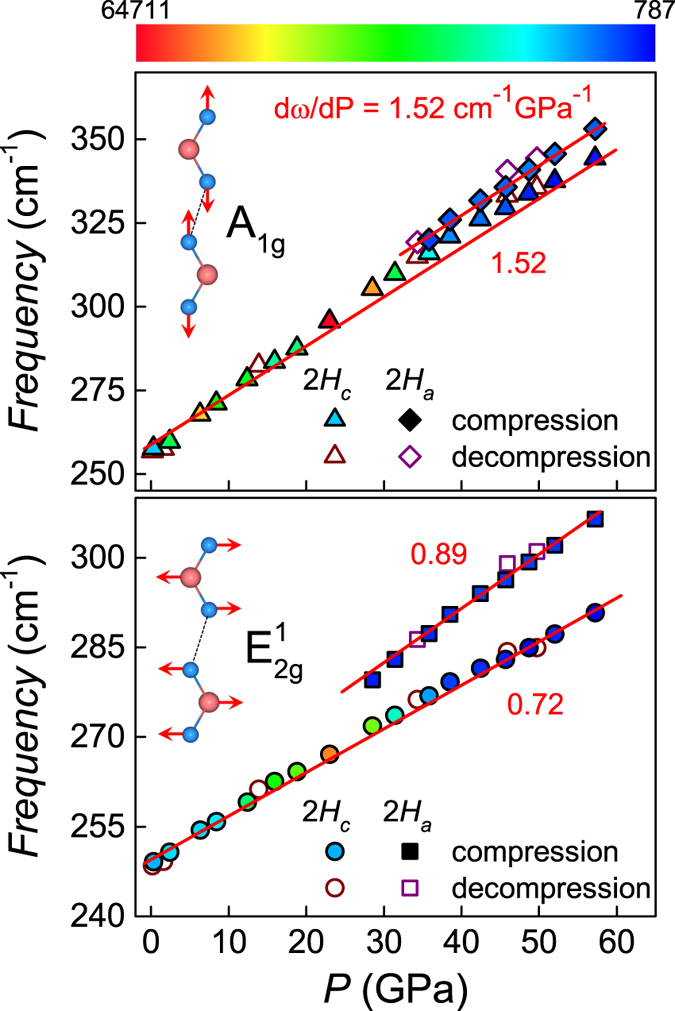
Peak frequencies of 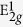 and A_1*g*_ modes as a function of pressure of WSe_2_, respectively. The solid lines are for eye guide. The peak intensity of modes is indicated by a colour scale extending from 787 (dark blue) to 64711 (bright red). The insets show the schematics of vibrational Raman modes with the black dotted line representing weak vdW bonds between the adjacent layers.

The pressure dependence of the frequencies of the 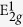 and A_1*g*_ modes are shown in Fig. 5. Upon compression, both modes show almost linear behaviors in the 2*H*_*c*_ phase, in roughly accordance with previous results to 20 GPa^23^. The slope of the 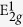 and A_1*g*_ mode is 0.72 cm^−1^ GPa^−1^ and 1.52 cm^−1^ GPa^−1^, respectively. As is known, the symmetry of WSe_2_ lattice can be described by the 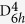 space group, and the irreducible representation of phonon modes at the center of the Brillouin zone is Γ = A_1*g*_ + 2 A_2*u*_ + B_1*u*_ + 2*B*_2*g*_ + *E*_1*g*_ + 2*E*_1*u*_ + *E*_2*u*_ + 2*E*_2*g*_, where the A and B modes are out-of-plane lattice vibrations, while the E modes are in-plane^32^. The 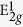 mode is from the vibrations involved in both W and Se atoms in the basal plane, with the opposite directions from each other. The A_1*g*_ mode refers only to the vibrations of the Se atoms along the *c*-axis (see the inset of Fig. 5). Consequently, the smaller slope of the 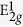 mode than the A_1*g*_ mode can be understood in terms of the anisotropic compressibility as the *c*-axis decreases much faster than the *a*-axis (see Fig. 3(a)). For the 2*H*_*a*_ phase, both modes increase linearly again but the slope of the 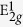 mode changes to 0.89 cm^−1^ GPa^−1^.

Upon approaching to the structural transition, the peak intensity of the A_1*g*_ mode increases slightly as can be seen from Figs 4 and 5 (color scale). Meanwhile, the intensity of 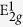 increases violently and then decreases rapidly at *P*_*C*1_ above which the iso-structural transition occurs. Outside the transition region, the peak intensity of both modes varies gradually with pressure. In addition, the slope of A_1*g*_ variation remains constant before and after the structural transition but the slope of 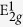 variation becomes larger from the 2*H*_*c*_ (0.72 cm^−1^ GPa^−1^) to 2*H*_*a*_ (0.89 cm^−1^ GPa^−1^). These observations imply that, while the out-of-plane compression is involved in the iso-structural transition due to anisotropic compressibility, the in-plane compression (strain) plays a dominant role in triggering the transition via layer sliding. This conclusion may explain the relatively low transition pressure (*P*_*C*1_ ~ 28.5 GPa) here compared with the previous theoretical calculations by only taking free mutual sliding of layers into consideration (~40 GPa)^21^,^24^.

**Figure 6:**
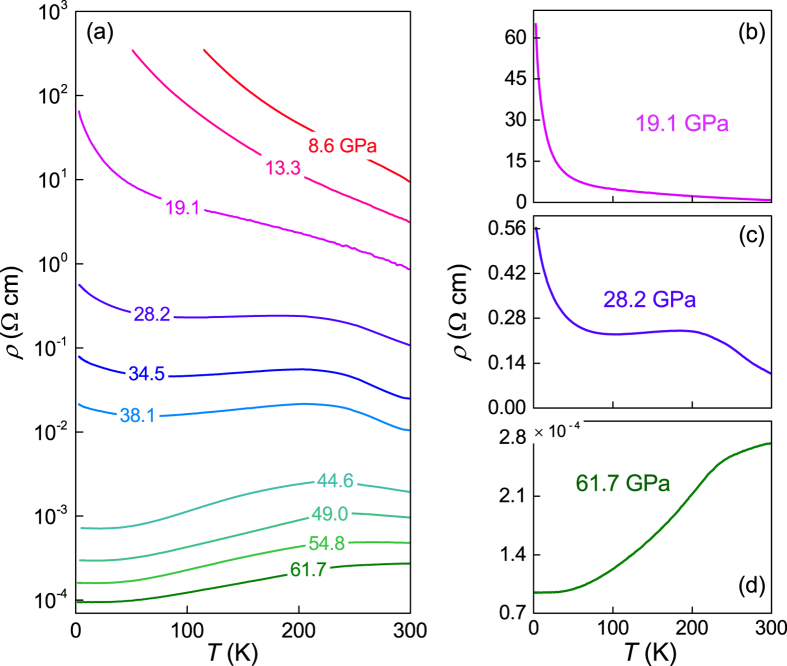
(**a**) The pressure dependence of resistivity curves of WSe_2_ as a function of temperature, with the maximum pressure up to 61.7 GPa. (**b**,**c**,**d**) The resistivity curves of WSe_2_ as a function of temperature at representative pressures: 19.1, 28.2 and 61.7 GPa, respectively.

We also performed *in situ* high-pressure resistivity (*ρ*) measurements for WSe_2_ in a diamond anvil cell up to 61.7 GPa. Standard four-point probe method was used. In order to get a more comprehensive understanding on the electrical properties than a previous investigation (290–420 K)^21^, we extended the temperature range down to 1.8 K. The results are displayed in Fig. 6(a). Roughly, two regimes can be identified upon compression: firstly, below 19.1 GPa, a typical semiconductor behavior (*dρ/dT* < 0) is observed; secondly, from 28.2 GPa to the highest pressure of 61.7 GPa investigated in this experiment, the resistivity first shows a plateau between 100 K and 200 K and then a metallic-like behavior (*dρ/dT* > 0), which becomes increasingly dominant. Figure 6(b)–(d) display the representative *ρ*-*T* curves for each phase. Our results clearly reveal a pressure-induced metallization for WSe_2_. In addition, the resistivity in the intermediate phase can be considered as a superposition of the low-pressure semiconducting and high-pressure metallic behaviors. These characteristics can be related to a coexistence of the low-pressure 2*H*_*c*_ and high-pressure 2*H*_*a*_ structures, in good agreement with the above XRD and Raman data.

## Conclusions

In summary, we have investigated the structural and electrical properties on single crystal WSe_2_. In spite of the lack of evident changes in the XRD patterns, the pressure dependent *c/a, θ* and *d*_*W*_ _−_ _*Se*_ consistently reveal two anomalies at *P*_*C*1_ ~ 20 GPa and *P*_*C*2_ ~ 40 GPa. The Raman active 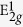 and A_1*g*_ modes split into two peaks, beginning at 28.5 GPa and 35.8 GPa, respectively, and lasting to the highest pressure of 57.2 GPa. These results strongly evidence that there exists an iso-structural transition for high-pressure WSe_2_. The distinctly different trend of peak intensity between 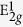 and A_1*g*_ with pressure suggests that the in-plane strain plays a dominant role in triggering the transition over the out-of-plane compression. Consistently, the resistivity measurements show a pressure-induced metallization, through a broad pressure range of 28.2–61.7 GPa, where the resistivity feature can be treated as a superposition of the low-pressure semiconducting and high-pressure metallic behaviors.

## Methods

WSe_2_ single crystals were grown via chemical vapour transport technique. High purity powders of tungsten (99%, Alfa Aesar) and selenium (99.99%, Alfa Aesar) with chemical stoichiometry were ground and sealed in an evacuated quartz ampoule (<10^−4^-torr vacuums). First, the components were made to react at 600 °C for 3 days. The loose sample was then re-ground and sealed in an evacuated quartz ampoule to get polycrystalline WSe_2_ samples. Second, an ampoule containing some polycrystalline WSe_2_ was placed in a two-zone furnace. The hot zone was maintained at 1060 °C and the cold zone was maintained at 1000 °C for 14 days. All weighing and mixing was carried out in a glove box. The crystal structure was characterized by room temperature XRD with Cu X-ray radiation (K_*α*_ = 1.5418 Å).

*In situ* high pressure synchrotron radiation XRD experiments were performed at 16-BM-D, HPCAT^33^ of Advanced Photon Source of Argonne National Laboratory using a Mao-Bell symmetric diamond anvil cell (DAC) with a culet of 300 *μ*m diameter. The as-grown single crystals were ground into fine powder for XRD experiments. A focused monochromatic X-ray beam (4.3 *μ*m (v) × 3.6 *μ*m (h) in FWHM) with a wavelength of 0.4246 Å was used for the X-ray diffraction experiment. The T301 stainless-steel gasket was pre-indented from a thickness of 300 *μ*m to ~35 *μ*m, and a center hole at 140 *μ*m in diameter was drilled to serve as the sample chamber. A pre-pressed powder sheet with a typical size of 30 *μ*m × 30 *μ*m × 5 *μ*m was loaded into the chamber together with a ruby ball^34^ and Daphne 7373 oil as pressure marker and pressure transmitting medium, respectively. The XRD patterns were collected with a Mar345 image plate detector. Refinements of the measured XRD patterns were performed by the GSAS software^35^.

Raman scattering experiments were carried out at the Center for High Pressure Science and Technology Advanced Research (HPSTAR) in Shanghai. The Raman spectrum measurement was performed using a commercial Renishaw Raman spectroscopy system with a 532 nm laser excitation line. The Raman system was first calibrated by using single crystal silicon with the characteristic peak at 520 cm^−1^ and the uncertainty is within 0.5 cm^−1^. The focused laser beam was about ~5 *μ*m. The laser power was 50% and the collection time was 100 s. Mao-Bell type DAC was used to generate the pressure. The measurements were conducted on a freshly cleaved single crystal (dimensions of 30 *μ*m × 25 *μ*m × 10 *μ*m). Daphne 7373 oil was used as the pressure medium. The diamond culet was 300 *μ*m in diameter. A rhenium gasket was pre-indented to a thickness of 29 *μ*m, and a center hole at 124 *μ*m in diameter was drilled. The pressure was determined by the ruby fluorescence scale below 20 GPa^34^ and the diamond Raman scale above 20 GPa^36^.

The pressure-dependent electrical transport experiments were performed by employing the standard four-probe method in a nonmagnetic Be-Cu cell. The diamond anvils is 300 *μ*m culets. A T301 stainless-steel gasket covered with a mixture of epoxy and fine cubic boron nitride (c-BN) powder was used for high-pressure transport measurements. Pt-foil (5 *μ*m) was used as the electrical lead. Standard four-probe method was applied in the *ab* plane of single crystals with typical dimensions of 90 × 40 × 10 *μ*m^3^. NaCl powder was used as the pressure transmitting medium. Pressure was calibrated with the ruby fluorescence shift at room temperature^34^.

## Additional Information

**How to cite this article**: Wang, X. *et al*. Pressure-induced iso-structural phase transition and metallization in WSe_2_. *Sci. Rep.* **7**, 46694; doi: 10.1038/srep46694 (2017).

**Publisher's note:** Springer Nature remains neutral with regard to jurisdictional claims in published maps and institutional affiliations.

## Supplementary Material

Supplementary Materials
