# Supplementary material for: Pressure-induced iso-structural phase transition and metallization in WSe2
Source: Sci Rep. 2017 May 4;7:46694. doi: 10.1038/srep46694 (PMC5415762; doi:10.1038/srep46694)
Supplement: Supplementary Materials [file srep46694-s1.doc]

Supporting Information for Scientific Reports

**Pressure-induced iso-structural phase transition and metallization in WSe_2_**

Xuefei Wang^1^, Xuliang Chen^1,*^, Yonghui Zhou^1^, Changyong Park^2^, Chao An^1^, Ying Zhou^1^, Ranran Zhang^1^, Chuanchuan Gu^1^, Wenge Yang^3,4^, and Zhaorong Yang^1,5,6,*^

^1^High Magnetic Field Laboratory, Chinese Academy of Sciences and University of Science and Technology of China, Hefei 230031, China

^2^High Pressure Collaborative Access Team, Geophysical Laboratory, Carnegie Institution of Washington, Argonne, Illinois 60439, USA

^3^Center for High Pressure Science and Technology Advanced Research (HPSTAR), Shanghai 201203, China

^4^High Pressure Synergetic Consortium (HPSynC), Geophysical Laboratory, Carnegie Institution of Washington, Argonne, Illinois 60439, USA

^5^Key Laboratory of Materials Physics, Institute of Solid State Physics, Chinese Academy of Sciences, Hefei 230031, China

^6^Collaborative Innovation Center of Advanced Microstructures, Nanjing University, Nanjing 210093, China

*Corresponding author, e-mail: xlchen@hmfl.ac.cn or zryang@issp.ac.cn


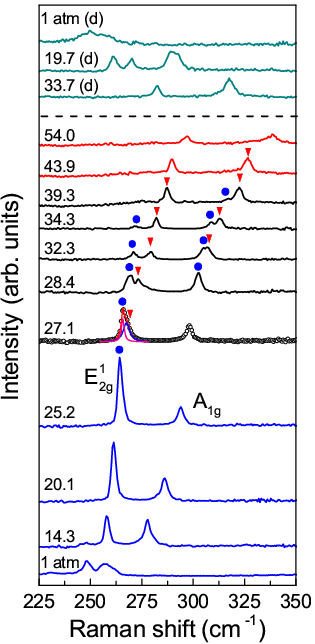


**Supplementary Figure 1.** Room temperature Raman spectra of WSe_2_ at selected pressures up to 54 GPa in both the compression and decompression (denoted by d) runs. The numbers represent pressures in unit of GPa. The dots and arrows indicate the appearance of splitting of the E_2g_^1^ and A_1g_ modes owing to pressure-induced phase transitions. At 27.1 GPa, solid lines are fitting to the experimental data by using the Lorentzian function.


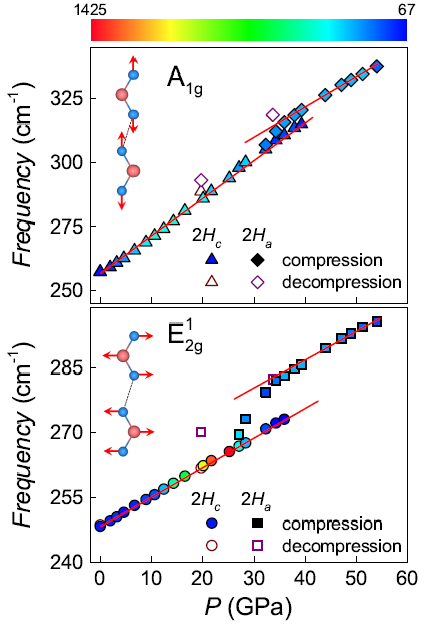


**Supplementary Figure 2.** Peak frequencies of E_2g_^1^ and A_1g_ modes as a function of pressure of WSe_2_, respectively. The solid lines are guided by eyes. The peak intensity of modes is indicated by a color scale extending from 67 (dark blue) to 1425 (bright red). The insets show the schematics of vibrational Raman modes with the black dotted line representing weak vdW bonds between the adjacent layers.

**Supplementary Note: Raman measurement**

Raman scattering experiments were carried out at High Magnetic Field Laboratory of Chinese Academy of Sciences. The measurement was performed at room temperature in a screw-pressure-type DAC made of nonmagnetic Cu–Be alloy using a Horiba Jobin Yvon T64000 spectrometer equipped with a liquid nitrogen cooled charge-coupled device. The Raman spectra were conducted in a pseudo backscattering configuration on a piece of freshly cleaved single crystal (dimensions of 50 µm × 30 µm × 10 µm) using 532-nm solid-state laser (torus 532, Laser Quantum) for excitation with a power below 0.1 mW. Daphne 7373 oil was used as the pressure transmitting medium. The diamond culet is 300 µm in diameter. T301 stainless-steel gasket was compressed from 250 µm to 30 µm, and a center hole of 130 µm was drilled to serve as the sample chamber. Ruby powders were placed aside the specimen to serve as the pressure marker^1^.

Representative Raman Spectra during compression and decompression (denoted by d) are shown in Supplementary Fig. 1. As the pressure is increasing, while the peak intensity of A_1g_ shows only a small variation, the peak intensity of the E_2g_^1^ mode increases significantly. At 27.1 GPa, the peak intensity of E_2g_^1^ reduces abruptly and the E_2g_^1^ mode begins to split into two peaks. Subsequently, a splitting of A_1g_ mode starts to appear at 32.3 GPa. Both the change of peak intensity and splitting of peaks evidence a pressure-induced iso-structural transition (Supplementary Fig. 2). Above 43.9 GPa, the feature of the structural phase coexistence seems not so obvious, namely, the Raman peaks from *2H*_C_ phase (blue solid dots) becomes almost indiscernible, which can be ascribed to a much lower signal-to-noise ratio in this run.

**Supplementary reference:**

1. Mao, H. K., Xu, J. & Bell, P. M. Calibration of the ruby pressure gauge to 800 kbar under quasi-hydrostatic conditions. *J. Geophys. Res.* **91**, 4673 (1986).
